# Unveiling Aortic Angiosarcoma: A Rare and Aggressive Vascular Malignancy in Vascular Oncology – A Case Report

**DOI:** 10.1177/15385744251355185

**Published:** 2025-06-21

**Authors:** Arnolda Marija Baškytė, Donatas Opulskis, Milda Kuprytė, Antanas Jankauskas, Linas Velička

**Affiliations:** 1 Department of Vascular Surgery, 230647 Lithuanian University of Health Sciences, Kaunas, Lithuania; 2 Faculty of Medicine, 230647 Lithuanian University of Health Sciences, Kaunas, Lithuania; 3 Department of Pathological Anatomy, 230647 Lithuanian University of Health Sciences, Kaunas, Lithuania; 4 Department of Radiology, 230647 Lithuanian University of Health Sciences, Kaunas, Lithuania

**Keywords:** angiosarcoma, hemangiosarcoma, primary aortic malignancy, open aortic surgery

## Abstract

**Introduction:** Primary aortic malignancy is a very rare pathology, with only 190 cases of aortic tumors reported in the literature to date. Usually, symptoms are insidious and nonspecific, so the disease is diagnosed at an advanced stage. **Objective:** Here, we present a case report of a primary malignant tumor of the aorta. In our case, the diagnosis was established using computed tomography angiography (CTA) and biopsy of indeterminate masses obtained during aortography. **Methods:** The patient underwent surgical resection of the affected aortic segment along with a tumor. Histopathological examination revealed the diagnosis of a primary malignant tumor of the aorta - angiosarcoma. Postoperatively, patient received adjuvant chemotherapy according to the standard treatment regimen for sarcoma. **Results:** One month later, postoperative CT of a chest, abdomen and pelvis was performed, revealing no evidence of metastases or pathological lymph nodes in the examined areas. **Conclusion**: Combined surgical and systemic therapies may improve overall survival.

## Introduction

Primary aortic malignancy is a very rare pathology, with only approximately 190 cases of aortic tumors described in the literature.^1,2^ Malignant aortic tumors can be classified according to their pattern of invasion into the aortic wall (mural, intimal and mixed) or according to their histological type (sarcoma, malignant fibrous histiocytoma, angiosarcoma and leiomyosarcoma).^3,4^ Angiosarcomas can also be reffered to as hemangiosarcomas or endotheliosarcomas. Angiosarcoma tends to develop in the inner lining of blood and lymphatic vessels, and both the thoracic and abdominal aorta are affected with similar frequency. It was established that men are more frequently affected than women, especially those over 60 years of age. This disease often manifests with atypical symptoms such as embolic phenomena (eg, acute limb ischemia, stroke, etc.), renovascular hypertension, or, in rare cases, spontaneous aortic rupture.^5^ Therefore, angiosarcomas diagnosis can be mistaken for non-malignant aortic diseases such as aortic aneurysm, atheroma, or mural thrombus.^6^ Diagnostic imaging, including computed tomography angiography (CTA), magnetic resonance angiography (MRA) or fluorine-18 fluorodeoxyglucose positron emission tomography (PET)-CT are required for the evaluation of angiosarcoma and its metastases.

However, since most diagnostic imaging findings are nonspecific, histological and immunohistochemical examinations are required for a definitive diagnosis of angiosarcoma and its metastases.^7^

The choice of treatment tactics and overall treatment success remain controversial. As with other sarcomas, surgery is considered the primary treatment for angiosarcoma. Primary goal of the surgery is complete resection of the tumor. According to the literature, in extreme cases, limb-sparing surgical procedures or amputations may be necessary as an additional treatment. Chemotherapy and radiotherapy remain important treatments for angiosarcomas and they can be administered both before and after the surgery. Neoadjuvant therapy doses are typically lower than those given postoperatively. Doxorubicin is the main chemotherapy drug used in the treatment of sarcomas, however, some medical centers use a combination regimen of mesna, doxorubicin, and ifosfamide (MAI).^8^

Here, we present a case report of primary aortic malignancy treated surgically.

## Case Report 

**Figure 1.**
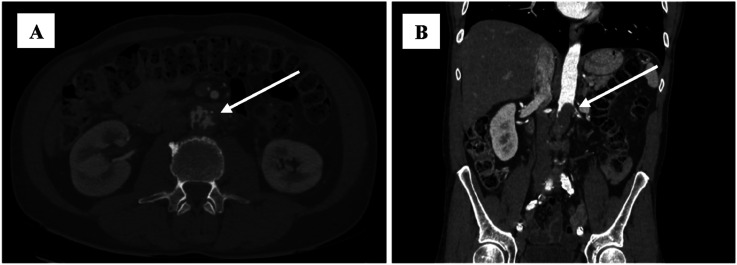
(A) and (B) CT angiography scan of the chest, abdomen and pelvis showing thrombus masses of unknown origin and atypical appearance in the abdominal aorta and left renal artery.

A 55-year-old male patient presented to the Hospital of Lithuanian University of Health Sciences Kaunas Clinics. At admission, the patient reported rest pain, burning and tingling senses in both feet, and a non-healing wound on the right foot. He also noted sleep disturbances due to acute ischemia of both limbs. The patient’s medical history revealed the onset of unknown origin spine and shoulder pain 9 months prior, which later progressed to involve both hip joints, leg muscles, and feet. On physical examination, patient appeared well-nourished with no signs of weight loss. The abdomen was non-tender, both feet were cool to the touch, microcirculation was slowed down, movements and senses were intact. Peripheral pulses were absent in the right popliteal artery (AP) as well as in the left dorsalis pedis artery (ADP) and posterior tibial artery (ATP). Laboratory investigations displayed elevated C-reactive protein (59.2 mg/L) and white blood cells (15.7 × 10*9/l) without clinical signs of infection or systemic inflammation. CTA of the abdomen, pelvis and legs revealed unevenly thrombosed, more than 50% narrowed infrarenal aortic lumen with abundant border thrombi (Figure 1).

**Figure 2.**
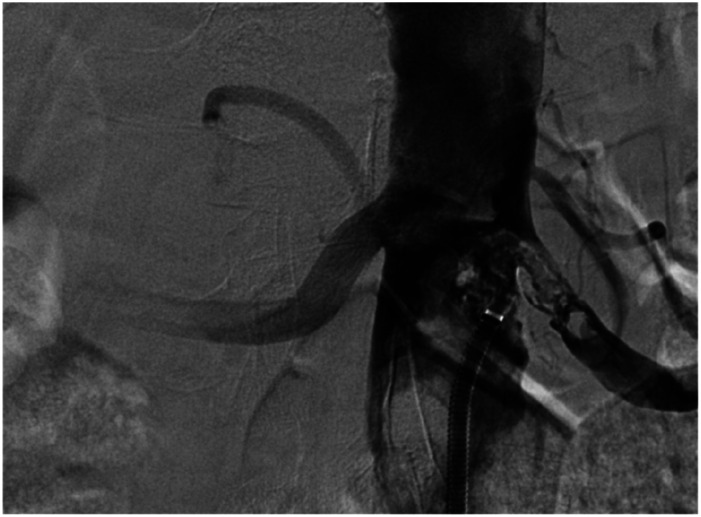
Aortography revealing a tumor mass, accompained by a biopsy sample for histopathological analysis.

The left renal artery was narrowed by 90% and sub-occlusion of the inferior mesenteric artery (IMA) was noted. In the right leg, the superficial femoral artery (AFS) was narrowed by 90%, and in the left leg, all major arteries of the calf were occluded. Due to unclear nature of the thrombotic masses of the abdominal aorta on CTA images, aortography was performed. The procedure was performed via the left common femoral artery, using a 7F introducer sheath. For diagnostic purposes, 5-6 biopsies of the thrombus mass were obtained using 105 cm length Argon Jawz™ endomyocardial biopsy forceps (Figure 2).

**Figure 3.**
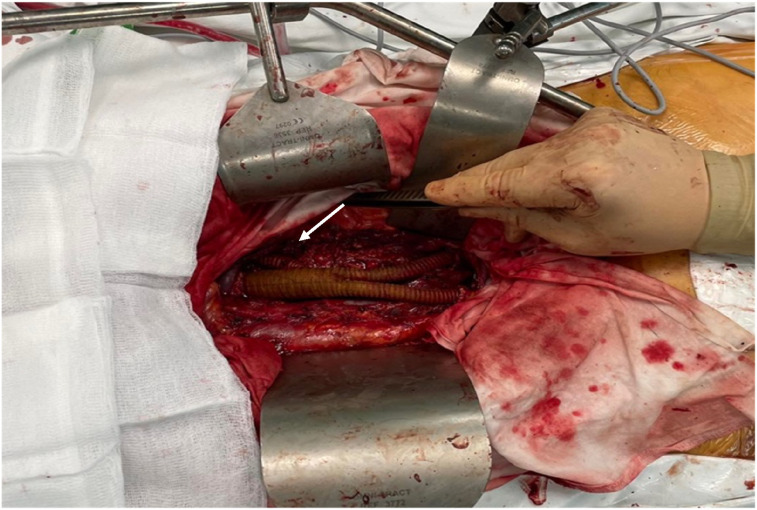
Intraoperative picture showing the interposition of a bifurcated graft at the level of the abdominal aorta, after resection of the angiosarcoma with a bypass to the left renal artery.

Urgent pathohistological examination of biopsy specimens revealed necrotic masses with unclear cell shadows, therefore, an oncological process could not be excluded. As the mass of unknown origin in the abdominal aorta had already caused emboli to both legs, malignancy was highly suspected and radical surgery was planned to prevent further thromboembolic events. In the first stage, the abdominal aorta was exposed through a full midline laparotomy. After aortic incision, gray, necrotic masses were found, raising a suspicion of an aortic tumor. Consequently, resection of the abdominal aorta along with the left renal and common iliac arteries was planned. Aortoiliac interposition with a left renal artery bypass was performed using a 20 × 10 × 10 mm vascular prosthesis (Figure 3 white arrow).

**Figure 4.**
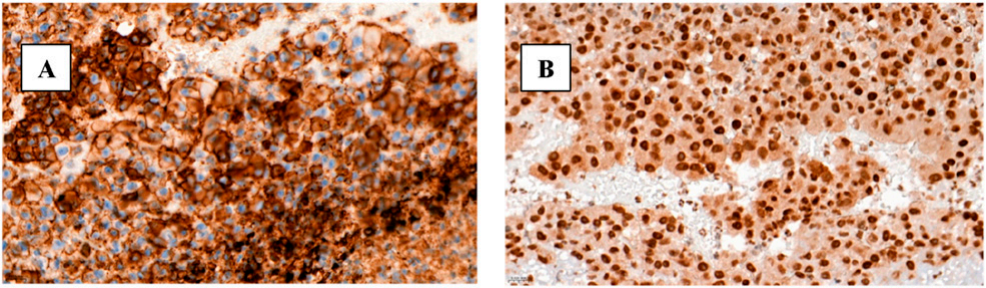
(A) Immunohistochemical reaction with antibody against CD31 (40x); (B) immunohistochemical reaction with antibody against FLI1 (40x).

Simultaneously, surgical thrombectomy was performed on both legs, and a large amount of thrombotic and tumor masses were obtained. On the first postoperative day, symptoms of Rutherford’s acute IIB ischemia developed in the left leg, prompting the decision to repeat surgical thrombectomy. The day following the second thrombectomy, due to the absence of clinical improvement and the progression of acute left leg ischemia, a below-knee (transtibial) amputation was performed. Postoperatively, the patient was prescribed a direct oral anticoagulant, specifically rivaroxaban at a dose of 10 mg daily. The patient was discharged on the seventh postoperative day. Histolopathological examination of the resected aorta with tumor masses revealed tumor cells lacking specific architectural tumor structures. The tumor cells consisted of polymorphic, chromatin-rich nuclei with nucleoli, mildly basophilic and very sparse cytoplasm, as well as sparsely infiltrating blood vessels (up to 95% of the total area of the tumor section). The tumor masses formed exophytic projections into the vascular lumen. Immunohistochemical analysis demonstrated positive staining for vimentin, CD31 and FLI-1, while no expression was detected for CK AE1/AE3, S100, PLAP, LCA, CD34 and factor VIII. Based on these findings, a diagnosis of intravascular epithelioid angiosarcoma was established (Figure 4).

Three months later, a below the knee amputation on right leg was performed. Both amputated extremities were subsequently sent for histopathological examination.

No evidence of tumor were found, however, dark thromboembolic masses were identified within the arteries of both legs. After surgery, the patient’s case was reviewed by the multidisciplinary team, and the decision to initiate adjuvant chemotherapy using doxorubicin and ifosfamide regimen was made. In total, six chemoterapy courses were administered. For the first course, chemotherapy drug doses were reduced by 20% due to the patient’s general condition. During the second course, the doses were optimized, and during the third course, duration was prolonged to 4 days. The remaining chemotherapy courses were administered according to the standard protocol (Table 1).

**Table 1.** Adjuvant Chemotherapy Scheme With Doxorubicin and Ifosfamide for the Treatment of Angiosarcoma.

Drug	Dosage	Route	Frequency
Doxorubicin	20 mg/m^2^	IV	1, 2, 3 days of cycle
Mesna	3 mg/m^2^	IV	1, 2, 3 days of cycle
Ifosfamide + mesna	3 mg/m^2^ + 3 mg/m^2^	IV	1, 2, 3 days of cycle
Mesna	3 mg/m^2^	IV	1, 2, 3 days of cycle

**Figure 5.**
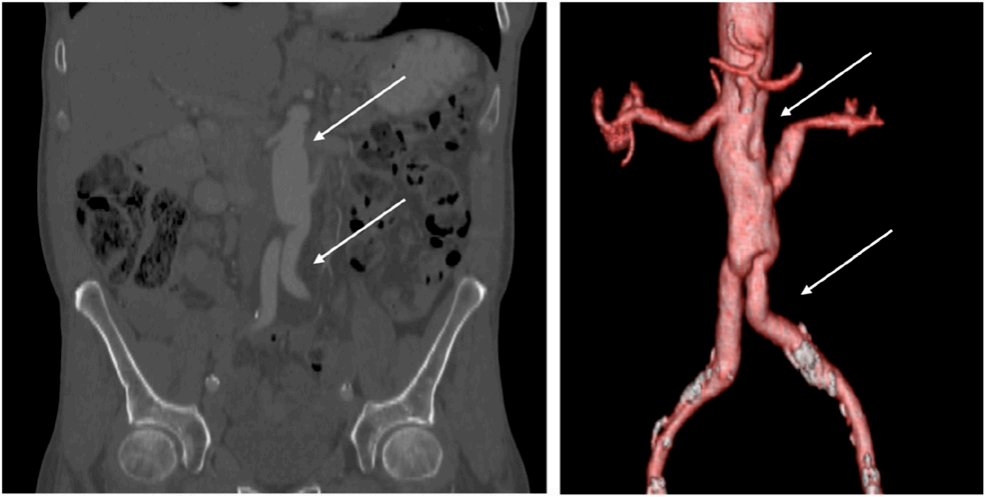
Control CT angiography and reconstruction of the chest, abdomen, and pelvis, which shows the vascular prosthesis. The proximal prosthesis end is sutured below the right renal artery, and both distal ends of the bifurcation to common iliac arteries (white arrows).

One month later, postoperative CT of the chest, abdomen and pelvis was performed, revealing no evidence of metastases or pathological lymph nodes in the examined areas (Figure 5).

## Discussion

Primary aortic angiosarcomas or sarcomas are a very rare type of neoplasia. Survival rates for angiosarcoma depend on the location and stage of the cancer. According to the literature, the median overall survival is 8 months, with an interquartile range (IQR) of 7 to 9 months. The reported survival rates are 26% at 1 year, 7.6% at 3 years, and 3.5% at 5 years.^9,10^

Based on the pattern of invasion and origin, tumors can be classified as intimal or mural subtype. Intimal angiosarcomas and sarcomas arise from the inner layer of the aortic wall - the intima - and most commonly present with thromboembolic events. Mural angiosarcomas and sarcomas originate from the middle layer of the aortic wall and the outer adventitia and are more often associated with local invasion.^11^ Our reported case corresponded to the intimal tumor subtype.

Although angiosarcomas and sarcomas usually involve the descending thoracic aorta, in our case, angiosarcoma affected the abdominal part of the aorta. These tumors tend to spread hematogenously, initially metastasizing to the lungs, following the liver, bones, soft tissues, and lymph nodes.^12^ No metastases to other organs were identified in our patient.

The clinical presentation is often characterized by nonspecific symptoms such as fever, asthenia, and thromboembolic events in the extremities, intraabdominal organs, or brain. Symptoms directly related to tumor growth, such as back pain or aneurysm rupture, usually occur in advanced stages of the disease, making timely and accurate diagnosis challenging.^13^ In the presented case, the patient developed bilateral lower limb thromboembolic events.

The prevailing opinion in the literature is that diagnosing a primary aortic malignancy requires a high degree of suspicion by the physician. For example, in cases of arterial thromboembolism with normal echocardiogram, and heterogeneously protruding aortic plaques on CTA images in the absence of generalized atherosclerotic disease, aortic sarcoma should be suspected.^6^ Kamran et al suggested that certain radiological imaging findings, such as protruding vegetations, a nodular soft tissue component, absence of atherosclerosis on CTA or neovascularization and a mass-like signal distinct from a mural thrombus on magnetic resonance imaging (MRI) should raise suspicion for primary aortic malignancy.^14^ Other authors emphasize that primary aortic angiosarcomas may have features like aortic thrombosis on CT images.^15^ However, a definitive diagnosis can only be made by pathologic examination, through biopsy sampling before surgery or pathological evaluation after surgical resection.

According to an article from the American Journal of Cancer Research, histologically, angiosarcomas are divided into well-differentiated and poorly differentiated types.^7^ In well-differentiated angiosarcomas, irregular vascular channels lined with endothelial cells are observed. In poorly differentiated angiosarcomas, in contrast, spindle-shaped, polygonal, epithelioid, and primitive round cells with increased mitotic activity and poorly formed vascular spaces are found. In the presented case, the primary aortic malignancy in the abdominal aorta appeared as an indistinct mass resembling thrombosis and histological examination revealed tumour cells lacking specific architectural tumour structures, with polymorphic nuclei, sparse cytoplasm, and abundant background necrosis - features consistent with poorly differentiated angiosarcoma described in the article. Immunohistochemistry is often useful in diagnosing poorly differentiated types of angiosarcoma. Angiosarcomas typically express endothelial markers, including factor VIII-related antigen (factor VIII), CD31, CD34, and vascular endothelial growth factor (VEGF). Among these markers, CD31 is found in more than half of the cases, therefore, because of its high sensitivity and specificity, is considered the gold standard for diagnosing angiosarcoma. In our case the tumour cells reacted positively with CD31, vimentin, and FLI-1, which supports the endothelial origin of the tumour. Interestingly, there was no expression of CK AE1/AE3, S100, PLAP, LCA, CD34, and factor VIII which is notable, as the article suggests factor VIII is commonly expressed in angiosarcomas. This discrepancy suggests an atypical or aggressive variant of poorly differentiated angiosarcoma.

According to the National Comprehensive Cancer Network (NCCN) guidelines, angiosarcomas are treated similarly to other soft tissue sarcomas.^16^ Surgical resection is recommended for patients with non-metastatic disease and should include the aorta with the tumor and any structures that appear to be related to the primary tumor. For metastatic disease, palliative chemotherapy with anthracyclines, ifosfamide and taxanes as well as radiation therapy are advised. The role of biological therapies remains under investigation.^10^

Our patient was diagnosed with nonmetastatic primary aortic malignancy and underwent surgery. Resection of the abdominal aorta, left renal artery, and common iliac arteries was performed, followed by an aortoiliac interposition and a left renal artery bypass. Adjuvant cytotoxic chemotherapy with doxorubicin and ifosfamide was administered postoperatively.

## Conclusion

Primary aortic sarcomas are rare malignancies that usually manifest in the advanced stage with limited treatment options. Combined surgical and systemic therapies may improve overall survival. However, further research is necessary to optimize early diagnostic strategies enabling timely radical removal of the tumor with the goal of achieving disease remission.
